# Operationalizing multisector partnerships: a Theory of Action and Reflection tool for zoonotic influenzas

**DOI:** 10.1093/heapol/czaf064

**Published:** 2025-09-10

**Authors:** Syed Shahid Abbas, Manish Kakkar, Gerry Bloom, Lewis Husain, Tim Shorten, Pushpa Ranjan Wijesinghe, Nilesh Buddha, Edwin Ceniza Salvador

**Affiliations:** Institute of Development Studies, University of Sussex, Brighton, United Kingdom; WHO Health Emergency Programme (WHE), World Health Organization Regional Office for South-East Asia (SEARO), New Delhi, India; Institute of Development Studies, University of Sussex, Brighton, United Kingdom; Institute of Development Studies, University of Sussex, Brighton, United Kingdom; Priory Farm, Half Moon Lane, Redgrave, Suffolk IP22 1RX, United Kingdom; WHO Health Emergency Programme (WHE), World Health Organization Regional Office for South-East Asia (SEARO), New Delhi, India; WHO Health Emergency Programme (WHE), World Health Organization Regional Office for South-East Asia (SEARO), New Delhi, India; WHO Health Emergency Programme (WHE), World Health Organization Regional Office for South-East Asia (SEARO), New Delhi, India

**Keywords:** multisector, intersectoral, collaboration, partnerships, integration, influenza, zoonoses

## Abstract

Zoonotic influenzas are major, ongoing public health policy challenge, not the least because of the importance of functional multisector partnerships (MSPs) for their prevention and control. However, despite years of investment in developing them, many countries have found multisectoral approaches, such as One Health, difficult to operationalize at national and subnational levels. One explanation for the lack of uptake is the limited nature of guidance on the design and adaptation of MSPs that consider local institutional dynamics. In this paper, we describe the process of developing a practical framework for assessment and characterization of MSPs. We use findings from an earlier review of academic and programmatic literature to develop a Theory of Action for multisector One Health partnerships that can nest into the short-term outcomes identified in the Theory of Change for One Health developed by the One Health Quadripartite. This comprises five elements: Characteristics; Starting conditions; Collaborative process; Outputs; and Responsiveness. We develop additional attributes to undertake a detailed characterization of different ‘levels’ of One Health partnerships. In addition, this Theory of Action allows for multiple outcomes of interest to be recognized and addressed. We then use the Theory of Action to develop a reflection tool to help country programme managers identify the specific characteristics of their respective One Health partnerships; recognize the differences in capacities and expectations of different partners; and use these insights to identify specific ways to strengthen the collaborative process. To our knowledge, this is the first time a detailed characterization of MSPs based upon programmatic attributes has been developed. Such a conceptualization of MSPs can facilitate the design, implementation, and evaluation of One Health and other multisector programmes and increase their relevance to the needs of the local context within which these are based.

Key messages

- There is a big know-do gap when it comes to implementing large multisector programmes at national and subnational levels. We propose a Theory of Action and Reflection tool that can help national programme managers identify their priorities and which can facilitate implementation of international guidance.
- Multisector programmatic initiatives can be assessed on the basis of the quality of their partnerships; their assessment does not need to rely on programme outcomes alone.
- We propose a reflection tool that can help practitioners and evaluators assess the design and function of multisector partnerships.

## Introduction

Zoonotic influenzas have high pandemic potential, having caused four pandemics over the past 100 years (Harrington *et al.* 2021). Ongoing outbreaks among wild birds, poultry and livestock have further highlighted their significance (Ly 2024). Zoonoses risks (Leach and Scoones 2013) are driven by developments in multiple sectors. This has led to calls for response strategies to include convergent approaches such as One Health (that highlights interdependence of animal, environment, and human health; Morse *et al.* 2012). However, despite their potential, multisector partnerships (MSPs), including One Health, remain difficult to define and operationalize.

While multisector responses often come together in the face of an emergency such as an outbreak, One Health remains difficult to implement in ‘peacetime’ (Halliday *et al.* 2012, Ripoll *et al.* 2022). Zoonoses response demands complex interactions of different sectors, disciplines, bureaucracies, and interests (Ebata *et al.* 2020). One Health partnerships are often fragmented due to the competing nature of incentives and understandings of problems and proposed solutions among the actors involved (Hinchliffe 2015). For instance, when it comes to addressing avian influenza, public health epidemiologists, poultry scientists, producers, and ecologists are likely to prioritize different interventions, such as personal protection, farm biosecurity, economic incentives for farming, and wetland management, respectively.

Unsurprisingly, limited consensus among the technical agencies responsible for defining a standard for multisector One Health partnerships (Abbas *et al.* 2022) is likely to compromise global governance as well as constrain the performance of in-country MSPs (Chien 2013, Lee and Brumme 2013).

Recognizing these challenges, international technical agencies, such as the Food and Agriculture Organization (FAO), World Health Organization (WHO), World Organization for Animal Health (WOAH), and United Nations Environment Program (UNEP), have requested better ‘guidance and tools for effective implementation of multisectoral approaches’ (FAO *et al.* 2022: 23). Accordingly, in this paper, we describe our efforts to develop a Theory of Action (ToA) for zoonotic influenza prevention and control that is nested within the recently proposed Theory of Change (ToC) from the One Health High Level Expert Panel (OHHLEP) (Dar *et al.* 2022). We propose to pilot this tool at country level for further testing and validation.

We focus on zoonotic influenza because of the urgency to respond to the ongoing influenza panzootic and reduce its pandemic potential. In this paper, we first review existing programme and academic literature to make a case for and propose a ToA for One Health response at national level. We also propose methods to characterize MSPs and to identify the processes needed to enact collaborative outcomes for in-country partnerships around zoonotic influenza. We then use the ToA to develop a reflection tool that can be used by researchers and practitioners to design, manage, and evaluate MSPs around zoonoses as well as potentially other complex challenges requiring a multisector response.

## Existing guidance and approaches to multisector partnerships

In this section, we outline some of the gaps in current operational guidance around MSPs which led us to develop a ToA and a practical reflection tool.

International technical agencies have developed a suite of practical guidance and operational tools in the past few years. This includes a suite of tools to assess sector-specific capacity for animal and human health as well as operational tools (Pelican *et al.* 2019, Behravesh *et al.* 2024).

However, despite their number, many MSP tools remain inadequate for use at national and subnational levels. This is because they often fail to incorporate the complex interplay of social, economic, political, regulatory, and ecological factors that shape effective multisectoral preparedness and response—both for zoonoses in general (Traore *et al.* 2023: 674) and for zoonotic influenza programs in particular (Mwacalimba and Green 2015; Chattopadhyay *et al.* 2018).

Because MSP members might include multiple, competing interests, it is difficult to identify simple outcomes of success that work for all (Errecaborde *et al.* 2019, Abbas *et al.* 2022). Therefore, programme managers require a flexible approach to design MSPs in a way that allows them to suit their local requirements. Accordingly, we propose a set of approaches in the following two sections on developing ToA for MSPs and a practical reflection tool that can complement existing guidance.

## Theorizing multisector partnership programmes

### Need for a Theory of Action

Programme theories explain how an intervention might contribute to intended or observed outcomes. Ideally, this should consist of both a ToC as well as a ToA. In the context of zoonotic influenza, the recently developed definition of One Health along with the related ToC by the OHHLEP offers an inclusive understanding of MSPs that accommodates multiple interests or outcomes for One Health. These include ‘collective need for clean water, energy and air, safe and nutritious food, taking action on climate change, and contributing to sustainable development’ (One Health High-Level Expert Panel (OHHLEP) *et al.* 2022).

These efforts to develop a programme theory that recognizes the place of non-health interests within MSPs are welcome. However, the related OHHLEP ToC does not clarify the process through which the high-level pathways of change (around policy, practice, and knowledge) might translate to practical outcomes for the global One Health approach (Dar *et al.* 2022)—which is reflective of implementation challenges faced by national influenza programmes (Mwacalimba and Green 2015).

A ToA, on the other hand, can explain ‘the way in which programmes or other interventions are constructed to activate these theories of change’ (Mayne 2017) and effect programmatic change. Accordingly, we developed a ToA based upon an earlier literature review on intergovernmental collaborations. Detailed methods and discussion for the critical review can be found in the full paper (Abbas *et al.* 2022). Key findings from the review which are relevant for this paper are summarized below.

### Elements of a multisector partnership

While most guidance around MSPs, especially around zoonotic influenza and pandemic preparedness, assumes that MSPs operate in formal settings, the review demonstrated that MSPs can assume a wide range of forms, including ad hoc and informal ones. Therefore, we concluded in the review that as an initial step in understanding MSPs, it will be helpful to ‘characterize’ them using descriptive qualities—such as scale, scope, formality, and strength. Such categorization will help provide a framework for programme managers to describe the unique nature of their respective MSPs.

Secondly, the review found that a complex systems understanding of MSPs is a helpful way to account for the presence of a large number of actors/components who are interconnected and influence each other through non-linear and dynamic modes of interactions (Zinsstag *et al.* 2012). Therefore, unsurprisingly, many experts use a complex systems framework to describe the ‘collaborative process’. They suggest that MSPs are initiated under a given set of ‘starting conditions’, such as sector failure and interdependence. The actual ‘process’ of working together is context dependent and has been referred to variously as collaborations or partnerships (Thomson *et al.* 2007, Emerson *et al.* 2012).

Finally, MSPs consist of actors who might have different expectations from the MSP. Their ‘preferred outcomes’ likely promote their individual interests over shared goals (Thomson *et al.* 2008, Tonelli *et al.* 2018). We also found that the commercial, economic, and political dynamics of zoonotic influenza-related MSPs have been discussed in academic literature (Bogich *et al.* 2012, Galaz *et al.* 2015), these have not always been addressed in operational guidance (Traore *et al.* 2023).

### Elements of Theory of Action

**Figure 1.**
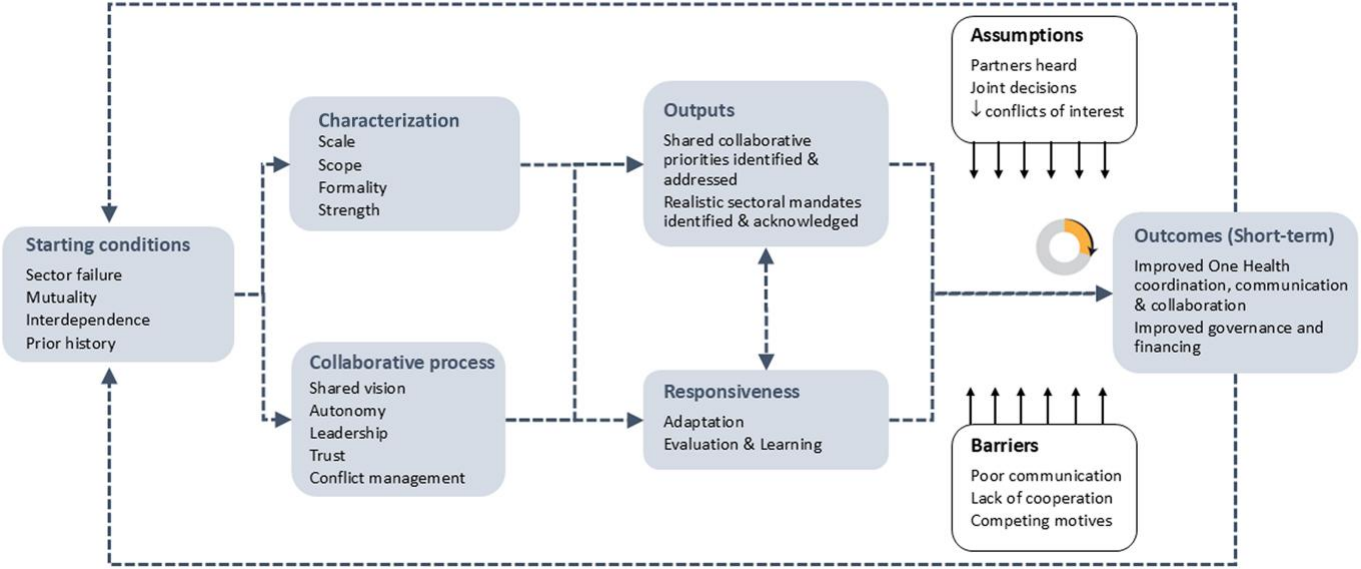
A Theory of Action for operationalizing multisector partnership.

The sequencing of elements, outcomes, and impacts matters for a ToA (Patton 1989). We used the review findings discussed above to identify elements of a ToA for MSPs around zoonotic influenza prevention and control. While the different elements in our theory are depicted in a linear relationship for the sake of simplicity in Fig. 1, the relationships between different elements are likely to be non-linear and interdependent. Given our overall aim of addressing the issue of MSPs related to the prevention and control of zoonotic influenza, we have chosen to restrict our focus to two of the six short-term outcomes identified in the OHHLEP ToC (coordination, communication, collaboration, equitable inclusion, governance, and financing) that were of most interest, namely, coordination and governance.

We then worked our way backwards from the *outcomes* in the OHHLEP ToC and populated the ToA using the four themes identified from our literature review (characteristics, starting conditions, process, and outcomes). We subsequently developed the description and connections between various elements through an iterative process, including consulting with academics and managers outside the core authorship for further robustness.

A brief description of the key elements is discussed below.

*Starting conditions*: MSPs rarely arise due to common goals. Instead, different actors come together under conditions of uncertainty, crisis, or sector failure—when no single sector has the knowledge or resources to address the challenge. MSPs with actors having complementary strengths and a positive history of working together are more likely to sustain (Tennyson 2004, Emerson *et al.* 2012).

*Characterization*: MSPs can differ in terms of their structure, scope, and functions. We offer four attributes—scale, scope, formality, and strength to help programme managers identify characteristics of their respective partnerships, and which will need to be factored into any implementation plans. The MSPs can assume varying levels of formality (hierarchy, institutionalization, etc.) which are usually a function of local bureaucratic cultures. MSPs could also assume different levels of strength, as we discuss below (Abbas *et al.* 2022).

*Collaborative process*: Sustaining productive partnerships requires a combination of different intangible qualities. These include leaders who are good facilitators, boundary spanners, and knowledge brokers (Quick and Feldman 2014). A feeling of trust, respect, and relative autonomy among the partners would ensure that the weaker partners are able to contribute to the discussions and that their priorities are also addressed (Brouwer *et al.* 2016, Hitziger *et al.* 2018). Finally, the institutional cultures, bureaucratic hierarchies, and relationships influence the nature of MSPs (Shearer *et al.* 2016, Emerson 2018).

*Outputs*: This element is one of the differentiating features of our ToA. Competing motives among the partners are one of the key barriers to MSPs. This is because while the other sectors might share health-related priorities, their operating incentives are different from health-protection goals (Hinchliffe 2015, Selter and Salloch 2023). For instance, while the public health sector is chiefly motivated by health protection aims, the livestock sector would be servicing food safety, biosecurity, as well as food production, livelihoods and animal welfare priorities in the context of zoonotic influenza prevention and control. Even though it might not be possible to address all non-health priorities—such as food production—within a One Health MSP, by identifying and acknowledging those priorities, we expect that managers can make the competing motives explicit and thus reduce any conflicts within individual sectoral (e.g. food production, economy, and livelihoods) and collaborative (health-protection) aims. Accordingly, the outputs from an MSP could be multiple, diverse, and catering to the interests of their respective stakeholders.

*Responsiveness*: As mentioned earlier, it is usual to specify indicators of success for MSPs that focus on technical programme-centric goals instead of quality of MSP itself (Errecaborde *et al.* 2019, Abbas *et al.* 2022). However, we propose that successful partnerships are those in which the partners find value and therefore can sustain over time.

We found the list of barriers and enablers within the OHHLEP ToC to be a useful reference for further contextualization of assumptions for individual MSPs.

## Reflection tool to design locally relevant multisector partnerships

Good programme theories for MSPs do not automatically translate to good practice (van Tulder and Keen 2018). Accordingly, having developed the ToA, we then used its elements to develop a reflection tool that can be used by programme managers to design and adapt an MSP to suit their needs.

### Scope

We developed a reflection tool to allow a programme manager of a national MSP platform for zoonotic influenza prevention and control to: identify the unique characteristics of their respective MSP; recognize the differences in capacities and expectations of different MSP members; and identify specific ways to strengthen the collaborative process. Such a reflection tool would be able to help practitioners and evaluators identify outcomes that are relevant to the institutional dynamics and ambitions of their respective MSP and identify appropriate strategies to achieve them. Further, we developed a typology of MSPs that allows programme managers to situate the findings of the reflection tool to guide management of zoonotic influenza prevention and control programmes. While the outcomes from the ToA should benefit response activities, we expect the ToA to be used in preparedness and inter-outbreak periods when programme managers have the opportunity for reflection.

The reflection tool and related typology of MSPs are not designed to develop an ‘objective’ depiction of partnership or to develop cross-country comparisons which are not useful to the countries themselves (Traore *et al.* 2023). Instead, we see the reflection tool to be used as a management device that improves the implementation of existing technical guidance on MSPs for zoonotic influenza prevention and control by encouraging localization and periodic reflections to ensure the processes and priorities of the partnership remain responsive to changing external circumstances as well as changing partner expectations.

Using the ToA (Fig. 1) as an organizing framework, the reflection tool has been structured around four key sections: ‘Characteristics’ (to elicit the uniqueness of the MSP for zoonotic influenza prevention and control); ‘Motivations’ (to distinguish between shared and individual interests of stakeholders that have a bearing on zoonotic influenza prevention and control); ‘Collaborative process’ (to identify the governance of collaboration for zoonotic influenza prevention and control); and finally ‘Sustainability’ (to engender learning processes within programmatic MSPs for zoonotic influenza prevention and control). The next paragraph describes the reasoning behind the development of the first and longest section of the tool. We used ideas from existing public administration conceptualizations of the collaborative process to develop the section of the tool around motivations and collaborative processes (Bryson *et al.* 2006, Thomson *et al.* 2007). See Supplement S1 for an overview of the reflection tool.

### Programmatic attributes: a typology of multisector partnerships

The idea of levels, or degrees, of multisectorality has been raised earlier as well (Harris and Drimie 2012). However, while many attempts to define the quality of MSP performance were either too restrictive or focused on narrow outcomes, others placed the burden of defining metrics for success on either a specialist evaluator or the programme managers themselves (Rüegg *et al.* 2018, FAO *et al.* 2022). We used the findings from the literature review to develop a typology of MSPs and which some of the authors subsequently tested across multiple country contexts (Abbas *et al.* 2022, Shorten *et al.* 2024).

**Figure 2.**
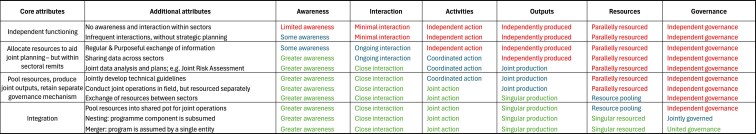
Additional attributes of MSP programmes (red, blue, and green colours correspond to low, medium, and high rank of each additional attribute, respectively).

With the aim of specifying the attributes that can be used by planners to define successful outcomes, we decided to further unpack the four levels of ‘strength’ of an MSP (Silo-based; coordination—sharing information; collaboration—joint operations in field; and integration—merger of assets) through programme-relevant ‘additional attributes’. These were: *Information* (awareness about other partner sectors); *Interaction* (frequency and mode of interaction); *Activities* (independently or jointly conducted in field); *Outputs* (independent or joint production); *Resourcing* (parallel or shared resourcing); and *Governance* (independent or united; Fig. 2).

**Figure 3.**
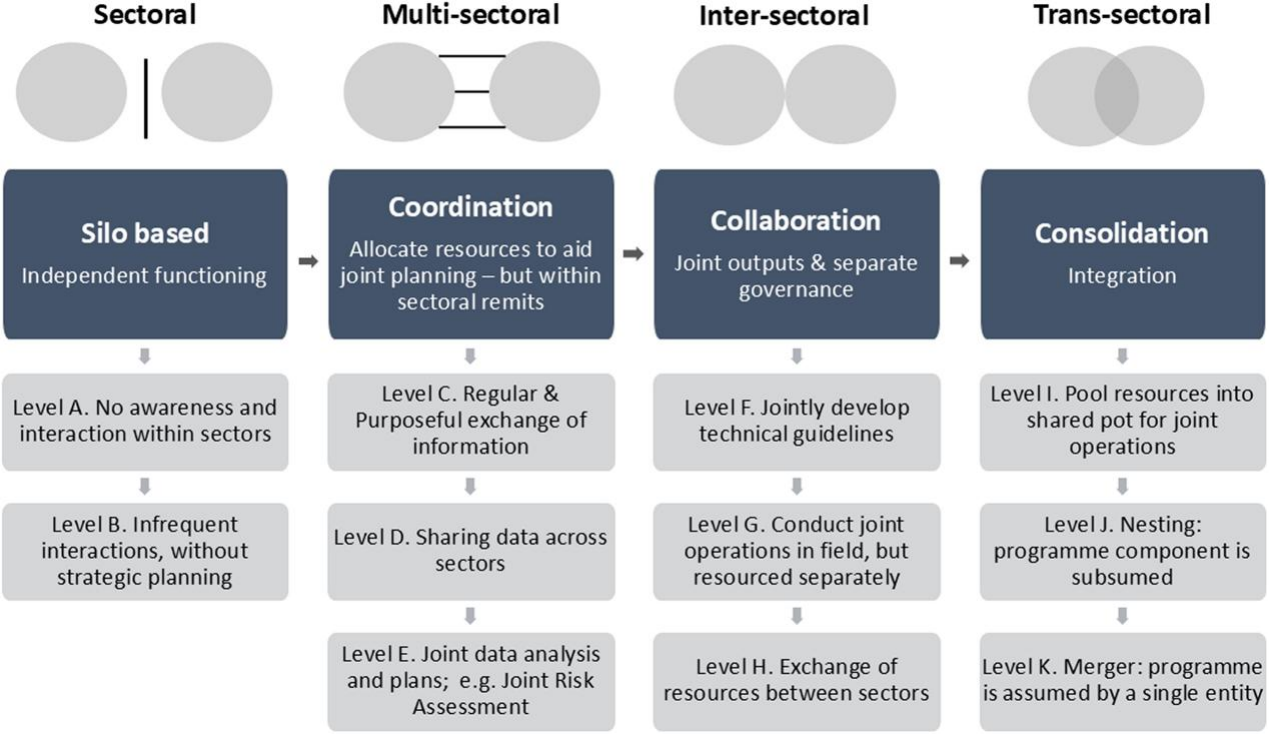
‘Levels’ of MSPs with varying strengths; adapted from Abbas *et al*. (2022, p.392).

These attributes will be given one of three ranks, based upon the definition in Table 1. Consequently, a combination of different attribute value would result in the scoring of one of 11 ‘levels’ of MSP, as depicted in Fig. 3. It is important to emphasize that there is no normative assumption implied within the different levels. We do not presume one level of MSP to be ‘better’ than another or that one level of partnership might be comparable with a similar level in another country context. Rather, we intend for this tool to be used by country programme managers to find out if their chosen level is ‘more relevant’ to their specific requirements, and even more importantly, use the additional attributes to help them plan how to achieve those levels of partnership.

**Table 1.** Definitions of additional attributes and ranks.

Additional attribute	Low rank	Medium rank	High rank
Information	Limited awareness Not much awareness about other sectors priorities	Some awareness Some understanding of the purpose, structure, and priorities of other sectors	Greater awareness Greater understanding of other sectors’ perspectives and concerns
Interaction	Minimal interaction No planned contact among officers of different sectors/organizations	Ongoing interaction Some planned meetings among officers of different sectors/organizations	Close interaction Regular and ongoing series of interactions, often accompanied with sharing of data/insights
Activities	Independent action Sectors acting independently of each other	Coordinated action Sectors apprise each other on activities of common interest	Joint action Sectors conduct field-level activities together
Outputs	Independent production Sectors produce outputs independently of each other	Joint production Sectors work on producing joint outputs (e.g. reports co-authored by ministries)	Singular production Outputs produced by a single, collaborative multisector entity (e.g. AMRCC or TWG)
Resources	Parallelly resourced Activities of each sector resourced from separate budgets/line items	Resource pooling At least some components of activities of one sector's activities resourced/subsidized from another sector, e.g. antiviral and medical screening of animal handlers provided through public health department in an AI outbreak	Singularly resourced Collaborative activities funded through the same shared pot
Governance	Independent governance Each sector reporting vertically to its line ministries	Joint governance Each sector team reporting to a joint entity, or when one component of programme is subsumed under a single entity while other components are governed independently	United governance All governance functions assumed by a single entity, e.g. AMRCC, National Pandemic Task Force, etc.

## Operationalizing multisector partnerships: bridging the gap between ‘what’ and ‘how’

This ToA and reflection tool was developed in response to the continuing challenge of weak MSPs in the prevention and control of zoonotic influenza and pandemic preparedness, particularly at national level (Traore *et al.* 2023). While the proposed ToA and reflection tool are grounded within the zoonotic influenza programmatic contexts, our methodology offers several insights that can be applied to other applications of MSPs as well. We enlist the key generalizable insights below.

There is a black-box approach to the governance of MSPs around zoonotic influenza as well as other contexts. Our ToA as well as reflection approach demonstrates that it is possible to assess function of MSPs on the basis of core MSP attributes (such as awareness, interaction, activities, and resourcing) without relying on overall programme outcome metrics (such as decreased disease risks) which are likely dependent upon other factors.

Moreover, in the context of zoonotic influenza prevention and control, the functioning of MSPs is grounded within local political dynamics. By including the acknowledgement of individual priorities such as public health, livelihood and trade as being important to the whole MSP, we hope to improve communications and reduce tensions that might arise from competing within MSPs.

It is important to reiterate that MSP are complex enterprises—each of them a product of different set of interests and compromises made in specific political contexts. Therefore, the purpose of our tools is not to promote cross-country comparisons or to prescribe which form of partnership is more desirable. Instead, we expect our tools to be used by local programme managers engaged in prevention and control programmes for zoonotic influenzas and other complex multisector challenges to help them identify the combination of attributes that are most relevant to their own institutional and programmatic needs.

Finally, we would like this reflection tool to be used to help with strategic management as well as evaluation approaches. This tool is not designed to be used for normative assessment or cross-country comparisons. Instead, our primary expectation from the tool is for it to help managers improve the functioning, effectiveness, as well as sustainability of their respective multi sector initiative; our secondary ambition is to facilitate development of practical and transparent evaluation approaches.

## Supplementary Material

czaf064_Supplementary_Data

## Data Availability

The data underlying this article are available in the article and in its online supplementary material.

## Reflexivity statement

The authors represent a wide range of geographic, disciplinary, and practical expertise as well as different seniority levels and geographic location. Four authors each are located in the UK and India, with the India-based authors working at a South East Asian region. The authorship includes expertise in health policy analysis (S.S.A., G.B., and L.H.), programme evaluation (T.S., L.H., S.S.A., G.B., and infectious disease epidemiology (M.K., P.R.W., N.B., and E.C.S.). The authors bring in different roles, including academic research (S.S.A., G.B., and L.H.), management expertise (T.S.), and technical advice (M.K., P.R.W., N.B., and E.C.S.).
