# Supplementary material for: Operationalizing multisector partnerships: a Theory of Action and Reflection tool for zoonotic influenzas
Source: Health Policy Plan. 2025 Sep 10;40(10):1142–8. doi: 10.1093/heapol/czaf064 (PMC12630573; doi:10.1093/heapol/czaf064)
Supplement: czaf064_Supplementary_Data [file czaf064_supplementary_data.docx]

# Supplementary File: Reflection tool

**Section I: Characterisation of Multi Sector Partnership**

1. Scope: What is the main function of your MSP? Choose ONE option that best reflects your MSP.

A. Produce policy reviews, provide technical advice to government institutions

B. Conduct activities in the field

C. Mostly work with individuals such as researchers, academics and technical experts - whose work is related to research and teaching

D. Select this if more than one of the above options apply to your MSP

Describe- If you can, please explain the scope your MSPs work in 1-2 sentences?

2. Scale: What is the jurisdiction of your MSP? In other words, which of the following administrative levels does your MSP collect information and / or design interventions for? Choose ONE option that best reflects your MSP.

A. Your MSPs work covers multiple countries.

B. Work with national / federal level agencies and ministries. Produce policies and oversee national level programmes across multiple provinces/states.

C. Work within a single province state.

D. Work at a district, local or community level

E. Select this if none of the options apply to you perfectly

Describe- If you can, please explain the scale your MSPs work in 1-2 sentences?

3. Formality: Which of the following three options best describes the way your MSP functions. Choose ONE option that best reflects your MSP.

A. Formal, rigid, institutional

The MSP has a fixed membership with mostly the same institutions / individuals represented at all meetings. It will be difficult to make changes to the membership and objectives of the MSP at a short notice.

The meeting schedules and agenda are (for the most part) decided in advance.

All discussions within MSP are through meeting notes, reports, etc. All interactions among individual MSP members are restricted within formal meetings or through official correspondence.

B. Official but informal, ad hoc and reactive

The MSP does not have a fixed composition or meeting schedule. Meetings take place whenever there is a need (outbreak, etc.) and all relevant individuals are called.

It is easy to call meetings at a short notice, if needed.

When required, the MSP members can call or email each other without going through official channels, including on social media such as WhatsApp.

C. Mixed: Some elements each from Option A and B

D. Describe- If you can, please explain the degree of formality within your MSPs work in 1-2 sentences?

4. Degree of Joining up

4A. Different MSPs find different ways of encouraging people to work together. In the case of your MSP, please identify the one statement that best captures most of your work with the MSP.

A. All MSP members work independently of each other most of the time

B.MSP members meet periodically, primarily to exchange information and ideas

C. MSP members conduct joint planning as well as implement activities together in the field

D. MSP members conduct joint activities as well as share common resources such as through cross-deputation of personnel and sharing office spaces

E. Describe- If you can, please explain the degree of integration within your MSPs work in 1-2 sentences?

5. Additional attributes

Think more carefully about the characteristics of your MSP. Select the option that appears to best describe the nature of your MSP.

5A. Information: How much awareness do other members have about your sector’s priorities and processes.

A. Limited Awareness - Not much awareness about other sectors priorities

B. Some Awareness - Some understanding of the purpose, structure and priorities of other sectors

C. Greater Awareness - Greater understanding of other sectors' perspectives and concerns

5B. Interaction: What is the nature of your interaction with members from the other sectors?

A. Minimal interaction - No planned contact among officers of different sectors/organisations

B. Ongoing interaction - Some planned meetings among officers of different sectors/organisations

C. Close interaction - Regular and ongoing series of interactions, often accompanied with sharing of data/insights

5C. Activities: What is the degree of awareness and coordination in the activities of different sectors?

A. Independent action - Sectors acting independently of each other

B. Coordinated action - Sectors apprise each other on activities of common interest

C. Joint action - Sectors conduct field-level activities together

5D. Outputs: What is the nature of partnership in the outputs produced by different partner sectors?

A. Independent production - Sectors produce outputs independently of each other

B. Joint production - Sectors work on producing joint outputs (e.g. reports co-authored by ministries)

C. Singular production - Outputs produced by a single, collaborative multisector entity (e.g. AMRCC or TWG)

5E. Resources: How is resourcing / funding coordinated within the MSP?

A. Parallelly resourced - Activities of each sector resourced from separate budgets / line items

B. Resource pooling - At least some components of activities of one sector's activities resourced/subsidised from another sector e.g. Antiviral and medical screening of animal handlers provided through public health department in an AI outbreak

C. Singularly resourced - Collaborative activities funded through the same shared pot

5F. Governance: What is the degree of integration in the governance of different partners sectors/organisations?

A. Independent governance - Each sector reporting vertically to its line ministries

B. Joint governance - Each sector team reporting to a joint entity, or when one component of programme is subsumed under a single entity while other components are governed independently

C. United governance - All governance functions assumed by a single entity, e.g. AMRCC, National pandemic Task Force, etc.

**Section II: Motivations**

According to you, what are the reasons for this MSP to develop? Use the following questions to identify your contributions and expectations from the partnership.

6. Mandates

What is the major mandate/purpose of your organisation? It does not have to relate to the MSP and feel free to list issues which might be outside the scope of the MSP. (Enlist a maximum of three)

7. Contributions

What are the three most important areas of strength which you/your organisation bring to the MSP?

8. Mutuality

Which are the major areas or challenges which you would look for help from other members of the MSP? Feel free to list challenges which might be outside the scope of the MSP. (Enlist a maximum of three)

9. Priorities

Which are the priorities for your MSP listed in your action plan? In case, you do not have an action plan, please list the main priorities identified for your MSP. (Enlist a maximum of three)

10. Prior history

10A. How will you describe the relationship between you/your organisation and those of other organisations represented in the MSP? Think of past instances of collaboration and tensions. (Select one response)

- Mostly positive
- Mostly negative
- Mostly neutral
- Not much history

10B. Think of your (or of a colleague’s) most recent interaction with one member of the MSP that readily comes to mind. What was the interaction about? Describe in 1-2 lines.

10C. Can you recall any positive experiences from that interaction? Describe in 1-2 lines.

10D. Can you recall any negative experiences from that interaction? Describe in 1-2 lines.

**Section III. Collaborative process**

11. Shared vision

11A. Do all the organisations within the MSP know what is expected from them? (Y/N)

11B. Have all the MSP organisations budgeted and allocated the resources needed towards the MSP and its activities? (Y/N)

11C. Describe- Are there some aspects of the MSP that some of the partners are unsure about? Please describe in 1-2 lines.

12. Autonomy

12A. Does the MSP hinder your organization from meeting its own organisational mission (e.g., food safety vs food security)?

12B. As a representative of your organisation, do you feel pulled between trying to meet both your organisation’s and the MSP’s expectations?

12C. If you answered yes to 11A or 11B, can you describe in 1-2 lines, the sources of these tensions?

13. Leadership & Coordination

13A. Is there a common mailing list, phone directory, or contact list for MSP members that MSP members have access to? (Y/N)

13B. Is there a designated coordinator / member secretary / chair who organises the agenda, invitations and meeting schedules? (Y/N)

13C. Describe in 1-2 lines, how is the leadership of this MSP organised? (e.g., rotating chairs, external leadership, or designated member secretary)

14. Conflict management

14A. In case of any conflicts, how would you think the MSP would resolve them? Select the most appropriate response.

- Would you resolve these at an individual level outside the MSP?
- Would you resolve them within the MSP?
- Are you likely to refer to an external authority (e.g., senior officer) to resolve the conflict?

**Section IV. Sustainability**

15. Adaptation

15A. Do you think the effectiveness of your own organisation’s operations have been positively impacted as a result of the MSP? (Y/N)

15B. Does your organization achieve its own goals better working with partner organizations in the MSP instead of working alone? (Y/N)

15C. Do you have space for modifying MSP objectives if needed in the future? (Y/N)

16. Evaluation & Learning

16A. Is there an evaluation strategy in place for the MSP? (Y/N)

16B. If you answered yes in 15A, does the evaluation strategy focus on outcomes that your organisation is directly interested in? (Y/N)
